# Drug-Induced Intersticial Nephritis. Clinical Case

**DOI:** 10.15388/Amed.2021.28.2.14

**Published:** 2021-08-11

**Authors:** Paulina Tekoriutė, Monika Matuliauskaitė, Laimas Virginijus Jonaitis

**Affiliations:** Faculty of Medicine, Lithuanian University of Health Sciences; Faculty of Medicine, Lithuanian University of Health Sciences; Department of Gastroenterology, Lithuanian University of Health Sciences

**Keywords:** mesalamine, interstitial nephritis, ulcerative colitis

## Abstract

5-Aminosalicylic acid (5-ASA) preparations are widely used in the treatment of inflammatory bowel diseases. The most commonly used medicine is mesalamine. Overall, it is a very safe drug with few side effects. A rare side effect of this medicine is interstitial nephritis (IN). With discontinuation of the drug the renal function usually restores. However, if damage has not been detected for a long time, irreversible changes may occur. In this article, we present a clinical case of mesalamine induced IN. A 56-year-old man who has had ulcerative colitis for 20 years, was admitted due to mesalamine induced acute renal failure. A year before, the patient had been diagnosed with mesalamine-induced interstitial nephritis and the treatment with mesalamine was discontinued. The symptoms of ulcerative colitis worsened, and the patient independently decided to start taking mesalamine, which resulted in worsening of his health condition and impaired renal function. Mesalamine has been discontinued, additional treatment for acute kidney failure has been administered including hemodialysis. Renal function recovered and the patient was released for further treatment of ulcerative colitis and monitoring of renal function.

## Abstract (lt)

**Medikamentų sukeltas intersticinis nefritas. Klinikinis atvejis:**

**Santrauka. **5-aminosalicilio rūgšties (5-ASA) preparatai yra labai plačiai vartojami uždegiminių žarnų ligų gydymui. Dažniausiai vartojamas medikamentas – mesalazinas. Tai yra labai saugus vaistas, turintis nedaug šalutinių reiškinių. Retas šio preparato sukeliamas šalutinis poveikis yra inkstų pažeidimas (intersticinis nefritas). Nutraukus vaistų vartojimą, inkstų funkcija dažniausiai pagerėja, tačiau ilgą laiką nenustatytas inkstų funkcijos sutrikimas gali sukelti negrįžtamus pokyčius. Šiame straipsnyje pristatome mesalazino sukelto intersticinio nefrito klinikinį atvejį. 56 metų vyras, opiniu kolitu sergantis jau 20 metų, kreipėsi dėl ūminio inkstų funkcijos nepakankamumo, sukelto 5-ASA (mesalazino). Prieš metus pacientui jau buvo nustatytas mesalazino sukeltas intersticinis nefritas ir gydymas šiuo vaistu buvo nutrauktas. Paūmėjus opiniam kolitui pacientas savarankiškai pradėjo vartoti mesalaziną – tai sukėlė savijautos pablogėjimą ir inkstų funkcijos sutrikimą. Pacientui taikytas gydymas hemodializėmis ir medikamentais, buvo teigiama dinamika, todėl jis išleistas tolesniam ambulatoriniam gastroenterologo stebėjimui ir gydymui.

**Raktiniai žodžiai: ** mesalaminas, intersticinis nefritas, opinis kolitas

## Introduction

Ulcerative colitis is a chronic inflammatory bowel disease characterized by diffuse continuous inflammation of colonic mucosa [1,2]. 5-ASA preparations are the most commonly prescribed class of drugs to manage ulcerative colitis. The most common medication is mesalamine, which is administered to achieve the remission for patients with mild to moderate form of ulcerative colitis. Majority of patients with ulcerative colitis are on long-term maintenance treatment with mesalamine. Overall, it is a safe drug with very few side effects. An uncommon side effect of this drug is kidney damage – interstitial nephritis [3]. According to the UK General Practice Research Database, the incidence of renal disease due to mesalamine use is 0,17 cases per 100 patients per year [4].

**Objective: **To present a rare clinical case of mesalamine-induced interstitial nephritis.

## Clinical case

A 56-year-old patient was hospitalized to the Hospital of Lithuanian University of Health Sciences with acute renal failure (ARF), hyperkalemia (6.5 mmol/l), uremia (35 mmol/l), creatinine increase (1070 µmol) and anuria.

**Figure 1.**
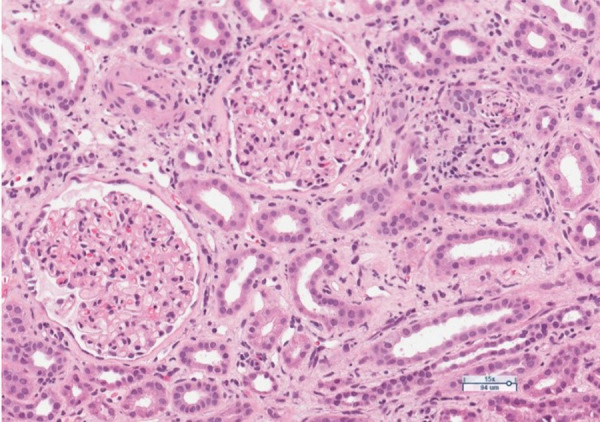
Limited foci of mixed inflammatory infiltrates with mononuclear leukocytes, plasma cells, neutrophils and eosinophils, with patchy fibrosis in the interstitium. Moderate arteriolar sclerosis. Glomeruli are unremarkable. H&E stain.

**Figure 2.**
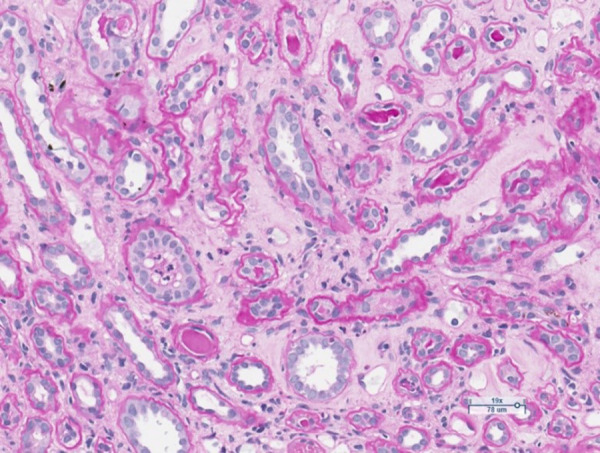
Limited foci of tubular atrophy and interstitial fibrosis. One tubule contains polymorphonuclear leukocytes. PAS stain.

The anamnesis revealed that the patient has had ulcerative colitis for 20 years and has been treated with short courses of mesalamine and no long-term maintenance treatment has been applied. Approximately 2 years ago, the azathioprine has been started as a maintenance treatment, but it was discontinued due to intolerable adverse events. The long-term maintenance treatment with mesalamine 2 grams daily has been administered. One year ago, patient developed severe acute renal failure and had been treated in the department of nephrology with the need of hemodialysis. At that time two renal biopsies were performed to clarify the etiology of ARF: the first one was noninformative, and in the second one, performed at the end of treatment inactive chronic interstitial nephritis was detected (Fig.1, Fig. 2).

The intake of mesalamine has been stopped and further treatment with mesalamine has been forbidden. At that episode, renal function parameters recovered and remained normal. 

One year later, due to exacerbations of the ulcerative colitis, patient independently started to use mesalamine 4 grams daily dose. After several weeks of the mesalamine intake, the general status of the patient worsened significantly. The patient developed weakness, stopped urinating, and began suffering from subfebrile fever. High uremic values were detected during the hospitalization: urea (35 mmol/l), creatinine (1070 µmol), GFR (5 mL/min/1.73 m2), potassium (6.5 mmol/l), hemoglobin level was 97 g/L, and there was no eosinophilia in the common blood investigation. Other blood and biochemical parameters were normal. Urine analysis was overall normal, with the exception of minimal leukocyturia in one urine specimen and it has been considered as not significant. Renal ultrasound showed structural, more intense, hyperechogenic renal parenchyma, and swollen kidneys: right kidney – 11.4 × 5.9 × 2.4 cm, left kidney – 11.8 × 5.7 × 2.1 cm. 

The patient was treated in the intensive care unit, where first hemodialysis has been performed. After 24 hours, the patient was transferred to the Nephrology Department for further treatment. Two more hemodialysis were performed. A decrease in uremic parameters was observed during the treatment. On the fifth day of inpatient treatment, the improvement of the patient’s condition was observed. As the uremic parameters kept decreasing (urea 12 mmol/l, creatinine 261 µmol/l) and the daily diuresis was 1300 the hemodialysis has been discontinued. 

The patient has arterial hypertension for some years. During his first episode of acute renal failure, the combination of Amlodipine, Hydrochlorothiazide and Olmesartan 20 mg/5 mg/12.5mg/ day, and Verapamil 240 mg daily has been taken. During the second episode he has been taking the same antihypertensive drugs as in the first episode plus Nebivolol 5mg daily. 

The patient was consulted by a gastroenterologist for the treatment of ulcerative colitis. Extensive ulcerative pancolitis was detected at endoscopy, with severe endoscopic signs of inflammation, endoscopic Mayo score – 3. Mayo score for ulcerative colitis provides an endoscopic assessment of disease severity. The score can range from 0 to3 with higher scores indicating higher severity (5). Histologically, active chronic ulcerative colitis was confirmed with a pre-existing or inactive cytomegalovirus infection. The prednisolone 40 mg/day has been administered orally for 2 weeks, later tapering the dose gradually 5 mg/weekly. Histologically suspected inactive or pre-existing cytomegalovirus infection was interpreted as clinically insignificant and therefore valganciclovir treatment was not prescribed.

As the condition of the patient improved, the patient was released from hospital for outpatient treatment and follow-up by gastroenterologist. The intake of mesalamine has been strictly forbidden. The tests conducted during the follow-up 2 weeks later showed a significant improvement – plasma creatinine concentration was 143 µmol/l, potassium – 4.41 mmol/l, urea – 10.8 mmol/l, 5 months after stopping the use of mesalamine, the patient’s glomerular filtration rate improve from 5 ml/min to 47 ml/min. The further treatment of UC with biological therapy (adalimumab) is planned.

## Discussion

There are various classes of drugs used for the treatment of UC: 5-ASA drugs (orally or locally), glucocorticoids systemically and locally, immunomodulatory (azathioprine or mercaptopurine orally) and other classes: calcineurin inhibitors (cyclosporine), biological therapy (monoclonal antibodies – anti-TNF-agents, anti-integrins, Janus kinase inhibitors) [2,6]. The most commonly prescribed 5-ASA drug is mesalamine, which can be administered orally or in the form of enemas, suppositories [1,6]. The safety and efficacy of sulfasalazine and mesalamine do not differ in this group, but sulfasalazine is worse tolerated, leading to increased use of mesalamine in clinical practice [1]. These medications have anti-inflammatory effect, they block cyclooxygenase, intestinal secretion of leukotrienes and other cytokines. About 28 percent of mesalamine enters the systemic blood circulation and is excreted by kidneys, therefore a dose adjustment is recommended based on renal function [7]. The usual dose is 2.0 to 4.8 g per day [1]. The patient under discussion received mesalamine 2 grams once per day until the first occurrence of acute renal failure. Before the second episode of renal failure the patient independently has been started to use 4 grams of mesalamine daily.

The course of interstitial nephritis can be acute and chronic. The classic form of acute IN is not dose related [7]. The most common symptoms are fever, rash, and lymphadenopathy. Laboratory tests may include pyuria and eosinophilia [7,8]. Another form of the disease manifests itself as a severe chronic, progressive, asymptomatic interstitial nephritis. It is thought to result from a cell-mediated response. The disease is difficult to recognize because it is asymptomatic and is most commonly found when blood creatinine and urea levels increase [8]. Kidney biopsy can show chronic IN in up to 58 percent of cases as it reveals chronic inflammatory changes in the interstitial tissue [7]. The biopsies from our patient also revealed the signs of interstitial nephritis. As the biopsy had been performed already sometime after the discontinuation of treatment with mesalamine, the low-activity chronic interstitial nephritis has been stated (Fig.1, Fig.2).

In the presented clinical case, after exacerbation of UC, the patient initiated mesalamine treatment and after a few days he developed fever, weakness, anuria, and acute renal failure with a signifi-cant increase in creatinine (1070 µmol/l). IN manifests in more severe forms among men rather than women. Mesalamine induced IN often occurs within the first year of treatment, usually within 4th to 48th months of treatment, but there have been several cases of IN where it occurred 5 years after initiation of mesalamine treatment [7]. Graham A. Heap et al. published a study which included 151 cases of 5-ASA induced nephrotoxicity. It was noted that 68 percent of nephrotoxicity cases occurred among men whose average age was 39.4 years and the median time of onset of renal damage at initiation of 5-ASA treatment was 3 years [9].

Theoretically it was possible, that the concomitant use of antihypertensive medications could act synergistically with mesalamine. Drugs interaction checker shows that hydrochlorothiazide, verapamil oral and mesalamine oral interaction is minor. Hydrochlorothiazide could increase the level or effect of mesalamine by acidic drugs competing for the same pathway through the kidney. Verapamil increases effects of mesalamine by unknown mechanism [10]. In our case, the medication administered for the treatment of arterial hypertension were not discontinued. As the renal function improved after the discontinuation of mesalamine, it is very unlikely that there was a significant pathogenetic role of the concomitant medications.

Renal function usually improves upon discontinuation of mesalamine, but glomerular filtration rate may remain reduced for a longer period of time. For 85 percent of the patients who discontinued mesalamine, renal function returned within 10 months after the beginning of the treatment. The improvement in renal function upon discontinuation of mesalamine was observed only in one-third of the IN patients who had received mesalamine for more than 18 months [8]. In the presented clinical case, 5 months after the discontinuation of mesalamine, the patient’s glomerular filtration rate recovered to 47 ml/min**. **It is generally recommended for tests to be performed to assess renal function 6 weeks before starting treatment with 5-ASA, 6 weeks from the beginning of treatment and thereafter – once a year [2].

As a conclusion we have to state that mesalamine may induce interstitial nephritis, therefore it is important to perform urine investigations and to monitor creatinine levels regularly during treatment with 5-ASA to prevent irreversible renal impairment. When nephrotoxic effects occur, it is necessary to discontinue the use of 5-ASA. Physicians should be aware of the possible rare side effects of 5-ASA medications, especially nephrotoxicity.
